# Obtaining subjects’ consent to publish identifying personal information: current practices and identifying potential issues

**DOI:** 10.1186/1472-6939-14-47

**Published:** 2013-11-25

**Authors:** Akiko Yoshida, Yuri Dowa, Hiromi Murakami, Shinji Kosugi

**Affiliations:** 1 Department of Medical Ethics/Medical Genetics, Kyoto University School of Public Health, Yoshida-Konoe-cho, Sakyo-ku, Kyoto 606-8501, Japan; 2 Department of pediatrics, Graduate School of Medicine, Kyoto University, Shogoin-Kawara-cho, Sakyo-ku, Kyoto 6068507, Japan; 3 Department of Clinical Genetics, Kyoto University Hospital, Shogoin-Kawara-cho, Sakyo-ku, Kyoto 6068507, Japan

**Keywords:** Consent, Consent forms, Identifying information

## Abstract

**Background:**

In studies publishing identifying personal information, obtaining consent is regarded as necessary, as it is impossible to ensure complete anonymity. However, current journal practices around specific points to consider when obtaining consent, the contents of consent forms and how consent forms are managed have not yet been fully examined. This study was conducted to identify potential issues surrounding consent to publish identifying personal information.

**Methods:**

Content analysis was carried out on instructions for authors and consent forms developed by academic journals in four fields (as classified by Journal Citation Reports): medicine general and internal, genetics and heredity, pediatrics, and psychiatry. An online questionnaire survey of editors working for journals that require the submission of consent forms was also conducted.

**Results:**

Instructions for authors were reviewed for 491 academic journals (132 for medicine general and internal, 147 for genetics and heredity, 100 for pediatrics, and 112 for psychiatry). Approximately 40% (203: 74 for medicine general and internal, 31 for genetics and heredity, 58 for pediatrics, and 40 for psychiatry) stated that subject consent was necessary. The submission of consent forms was required by 30% (154) of the journals studied, and 10% (50) provided their own consent forms for authors to use. Two journals mentioned that the possible effects of publication on subjects should be considered. Many journal consent forms mentioned the difficulties in ensuring complete anonymity of subjects, but few addressed the study objective, the subjects’ right to refuse consent and the withdrawal of consent. The main reason for requiring the submission of consent forms was to confirm that consent had been obtained.

**Conclusion:**

Approximately 40% of journals required subject consent to be obtained. However, differences were observed depending on the fields. Specific considerations were not always documented. There is a need to address issues around the study objective, subjects’ right to refuse consent and the withdrawal of consent. Whether responsibility for ensuring that the consent form has been signed lies with publishers also needs to be discussed.

## Background

Case reports providing new findings and identifying rare diseases play an important role in the development of medicine. Case reports improve medical practice and contribute significantly to medical education [1,2]. Some of them create a basis for clinical research [3]. However, because personal information is published in some studies and case reports, unlike large-scale clinical research, complete anonymity of personal information can be difficult to achieve [4,5]. Therefore, the International Committee of Medical Journal Editors (ICMJE) [6] and Committee on Publication Ethics [7] recommend proof of consent before publication of such information.

Obtaining research subjects’ consent has been regarded as problematic in some case reports, such as those containing pedigrees [8,9] or involving individuals with a questionable capacity to give consent, such as children and people with mental disorders [10]. Some journals provide their own consent form, and require the signed form as part of the submission process. Additionally, the issue of repeatedly having to obtain consent when journals accepting only their own consent form may reject a manuscript has been discussed [11,12]. The issue of the appropriateness of the requirement to submit signed consent forms–and therefore the subject’s name and health information–to the publisher has also been raised [13].

Previous studies have discussed the need to obtain consent [5,10,14-18] and have reported on journal consent forms and requirements for their submission [11-13,19]. However, current journal practices around specific points to consider when obtaining consent*,* the content of consent forms, and how consent forms are handled in case reports and other studies publishing identifying information have not yet been fully examined. This study was conducted to identify potential issues surrounding consent to publish identifying personal information.

## Methods

The study had two elements: a review of academic journals’ instructions for authors and consent forms, and a questionnaire survey of journal editors.

### Review of journal instructions for authors and consent forms

An Internet-based review was conducted of 545 academic journals in the fields of medicine general and internal, genetics and heredity, pediatrics, and psychiatry, as classified in *Journal Citation Reports* (2010). These four categories were selected to examine the general status of consent issues (medicine general and internal), because of problems regarding pedigrees [8,9] (genetics and heredity), because of problems in obtaining consent from guardians [10] (pediatrics), and because of difficulties surrounding consent in psychiatric diseases [5,10,20] (psychiatry). Of these journals, seven covered two fields. Instructions for authors that were available on the journal websites, written in English, and available between August 13 and December 13, 2011, were studied. Although web pages directly linked to these instructions were also studied, the contents of the rules themselves were analyzed in more detail. Content analysis focused on descriptions of consent for the publication of identifying information; identifying information was defined as information specified as such in the instructions for authors and information that allows the identification of subjects, such as information contained in case reports, photographs, pedigrees, addresses, initials, and names. Data available in the Cochrane Database of Systematic Reviews were excluded.

Consent forms developed by the journals and downloadable from their websites were also collected. When a journal’s instructions for authors mentioned that such a form was available upon request, the form was requested.

Content analysis of instructions for authors and consent forms was performed by three of the authors. AY initially analyzed all the data, and presented the results with text containing the basis of judgment. YD and HM read all the information included in the analysis and checked the result of first analysis independently. Any disagreements in the analytical process were discussed and resolved by all three authors. No software was used in this analysis.

### Questionnaire survey of editors

A questionnaire survey was conducted from August to October 2012 among editors of journals that require the submission of consent forms. The questionnaire was accessible on our website, and editors were asked by e-mail to respond to it. The type of editor (editor-in-chief or associate editor for example) was not specified, and the questionnaire was not anonymous. A single e-mail reminder was sent to each editor. This study was conducted with the approval of the Ethics Committee of Kyoto University.

The first part of the questionnaire concerned the time at which submission of consent forms was required, how long they were stored, and reasons given for requiring them. The next part consisted of questions on whether the journal provided its own consent form and whether it accepted other forms. If the journal did not provide its own form, respondents were asked what specific consent items were required [see Additional file 1].

## Results

### Review of instructions for authors

We conducted a review of instructions for authors to examine the journals’ policies on publication of identifying personal information. A total of 545 journals (153 for medicine general and internal, 156 for genetics and heredity, 108 for pediatrics, and 128 for psychiatry) were initially selected for study. Of those, 54 whose instructions for authors were not available online or not written in English were excluded, leaving 491 journals (132 for medicine general and internal, 147 for genetics and heredity, 100 for pediatrics, and 112 for psychiatry) to be reviewed.

Of these journals’ requirements for studies publishing identifying information, 203 (41.3%) included descriptions of consent or consent forms. The need to obtain consent particularly for the publication of pedigrees was mentioned by 36 journals (16 in medicine general and internal, 3 in genetics and heredity, 9 in pediatrics, and 8 in psychiatry). Substitution of consent by a relative or guardian in cases of individuals who cannot legally give consent – such as minors, individuals with impaired judgment, and the deceased – was mentioned by 93 journals (18.9%). The submission of consent forms was required by 154 journals (31.4%), and consent forms designed by the journal were available online or by request from 50 journals. Additional details on these results are provided in Table 1.

**Table 1:** Journals’ general instructions for authors on consent

	**Medicine general and internal journals**	**Genetics and heredity journals**	**Pediatrics journals**	**Psychiatry journals**	**Total**
Obtaining of consent/consent form is required.	74	31	58	40	203
	(56.1)	(21.1)	(58.0)	(35.7)	(41.3)
Substitute consent is accepted, including by a relative or guardian on behalf of a minor, person with impaired judgment, or person who has died.	27	16	29	21	93
	(20.5)	(10.9)	(29.0)	(18.8)	(18.9)
Submission of consent forms is required.	57	21	49	27	154
	(43.2)	(14.3)	(49.0)*	(24.1)**	(31.4)
Consent forms are provided by the journal.***	20 [3]	8 [0]	13 [3]	9 [2]	50 [8]
	(15.2)	(5.4)	(13.0)	(8.0)	(10.2)

*Notes:* Percentages are given in parentheses. A total of 491 journals’ instructions to authors were reviewed.

*One journal required approval by an ethics committee or consent forms signed by subjects for all case reports.

**One journal required the submission of consent forms after anonymization.

***The first number indicates the total number of journals providing consent forms; most make these available on their websites. The number in square brackets indicates the number of journals that provide the forms only in response to a request to their editors for these forms.

Journal instructions for obtaining consent were also examined (Table 2). In 37 journals (7.5%), authors were required to offer subjects an opportunity to review the manuscript before submission. Instructions regarding consent for non-paper-based publication (distribution online and through other media) were found in 21 journals (4.3%); of these, one mentioned an open-access license, while another instructed authors not to use photographs in manuscripts to be published online without obtaining consent for electronic publication. Permission for reproduction was addressed by three journals. Instructions for considering the effects of subject identification were found in the instructions of two journals, one of which instructed authors to pay attention to the possibility that children may have future regrets about the publication of their identifying information, even if their parents consented to it at the time. One journal required statements about submission of signed consent forms as part of the consent form. This journal required the inclusion of a statement that subjects’ identifying information would be disclosed to publishers with signed consent forms, to comply with the US Health Insurance Portability and Accountability Act, which protects patient confidentiality. This journal also specified that a relevant ethics committee’s written confirmation that consent had been obtained could substitute for the actual signed consent forms.

**Table 2:** Journals’ specific instructions for authors on consent

**Descriptions regarding the content of consent**	**Number of journals giving the instruction**
Subjects allowed to review the manuscript before submission	37
Permission for non-paper-based publication (distribution online and through other media)	21
Permission for reproduction	3
Statement regarding the potential effects of subject identification	2
Statement that signed consent forms will be submitted to publishers	1
**Consequences if consent forms are not provided**	
Anonymizing or removing identifying information contained in the article, or not publishing it	86
Independently considering the possibility of publishing the article without consent forms	8*
Requiring a statement giving reasons for the unavailability of consent or the inappropriateness of obtaining it	4
Requiring a statement giving reasons for the unavailability of consent and confidentiality procedures	2
Requiring the submission of documents specifying the responsibility of an ethics committee, clinicians, or people in equivalent positions for the article’s publication	2

*Note:* A total of 491 journals’ instructions to authors were reviewed.

*Seven journals left open the possibility of publishing such manuscripts without relatives’ consent when it is difficult to reach them after subjects’ deaths.

A sample consent form was provided by one journal, consisting of the subject’s or a guardian’s signature, date of consent, and statements about deletion of the subject’s name, permission for reproduction, materials used in the manuscript, and the authors’ names. Four journals accepted authors’ consent forms instead of their own. Statements about rejection or influence on reviews as possible consequences of submitting manuscripts without consent forms were found in the instructions for authors of four journals.

Instructions for removing identifying information when consent is not obtained from subjects or their substitutes were found in 86 journals (17.5%). These included statements such as “if subjects’ identifying materials are to be used, either they should be made non-identifying or the author should submit consent to use them” and “identifying information should not be published in a manuscript unless the subject gives consent for publication”. Eight journals stated that they would consider the possibility of publishing manuscripts without consent forms; of these, seven stated that one of the circumstances under which they would consider this is when it is difficult to reach a subject’s relatives after his or her death. Four journals required that authors provide reasons for the unavailability of consent or inappropriateness of obtaining it. Two mentioned the possibility of publishing manuscripts without consent forms if the authors provide a letter from relevant ethics committees, clinicians, or people in equivalent positions. Two other journals instructed authors to specify reasons for the unavailability of consent and methods to ensure anonymity (Table 2).

One journal stated that substitute consent is not valid for subjects with impaired judgment, as the benefits to them are unclear or uncertain. Similarly, seven journals belonging to the same publishing group stated that their editors avoid using photographs when consent for publication is questionable for patients with a mental disorder or learning difficulty.

### Review of journals’ consent forms

Consent forms developed by journals and available on their websites were collected and examined. A total of 27 consent forms from 42 journals (17 for medicine general and internal, 8 for genetics and heredity, 10 for pediatrics, and 7 for psychiatry) were studied, five of which were used in multiple journals belonging to the following groups: British Medical Journal publishing group (7 journals); The journal of the American Medical Association (4); BioMed Central (4); Ovid (3); and Adis (2). Although eight journals stated that consent forms were available from their editors, and requests for forms were made to those journals, there were no replies.

All consent forms required the signature of the subject or a substitute. Of the 27, 26 clearly mentioned their online or paper-based status, and six stated that online journals are published freely or not freely. Difficulty in completely anonymizing subject data was addressed by more than 80% of all consent forms, seven of which showed examples of this difficulty. Two required additional consent for inclusion of potentially identifying photographs in a manuscript even in the absence of a subject’s name or initials.

**Figure 1:**
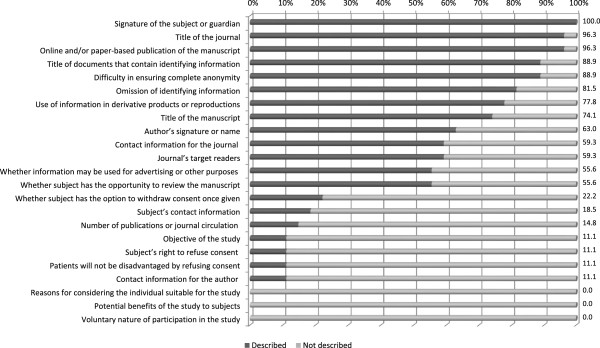
Items included on consent forms provided by journals.

Statements regarding use of information in derivative products or in reproductions and reprints, the title of the manuscript, and authors’ names and contact information were found in 59.3% to 77.8% of journal consent forms. Statements regarding the journal’s target readers were found in 16 of the forms, 15 of which mentioned the possible availability of manuscripts in printed, online, or derivative products to nonmedical professionals and general readers throughout the world. Statements regarding the use or non-use of data for other purposes, including advertising, were found in over half (55.6%). The possibility of advertising use was ruled out in 11 forms and affirmed in four. The subject’s opportunity to review the manuscript was addressed in 15 (55.6%), three of which also provided a place for subjects to indicate whether they wanted to review it. The possibility of withdrawing consent after signing was addressed in six forms (22.2%), three of which allowed withdrawal before publication and three of which did not. The study’s objective, subject’s right to refuse consent, and assurance that patients would not be disadvantaged by refusing consent for publication of their information were stated in only a few consent forms, and none of them included a statement on inclusion criteria, potential benefits for subjects, or the voluntary nature of participation (Figure 1).

Twenty-one required submission of consent forms in instructions for authors; however, only 10 of these included statements about submitting signed consent forms to the publisher in their consent forms, while one specified, both in its instructions for authors and its consent form, that forms were not to be submitted to the publisher. Some consent forms required additional permission for online reproduction and publication. Five were available in multiple (2 to 14) languages.

### Questionnaire survey of editors

To investigate the management of signed consent forms, editors of journals with instructions for authors that included a requirement for the submission of consent forms were asked to participate in an online questionnaire survey. Excluding three journals covering two fields and one without an e-mail contact address from the 154 targeted journals, the request was sent to editors of 150 journals. The response rate was 15.3% (23 responses; 12 in medicine general and internal, 1 in genetics and heredity, 7 in pediatrics, 2 in psychiatry, and 1 representing multiple journals).

Questionnaire responses indicated that 18 journals (78.3%) required consent forms with signatures on submission of a manuscript. Submitted consent forms were stored indefinitely by 13 journals, four of which stored electronic versions. The most frequent reason for requiring the submission of consent forms was to confirm the receipt of consent (Table 3).

**Table 3:** Editors’ reports on their journals’ management of consent forms

**Questions**	**Number of responses**	**Percentage**
Time to submit signed consent forms		
When manuscript is submitted	18	78.3
When manuscript is accepted	2	8.7
After manuscript acceptance and before publication	1	4.3
Other	2	8.7
Period of storage of signed consent forms		
Until the obtainment of consent is confirmed	2	8.7
Until the manuscript is formally accepted or rejected	0	0.0
Until the manuscript is published	2	8.7
Until a certain period of time after publication*	6	26.1
Indefinitely**	13	56.5
Reasons for requiring submission of consent forms (multiple answers possible)		
To confirm the receipt of written consent	21	
To confirm the contents of the consent form	11	
Other	3	

*Note:* Survey responses represented 23 journals.

*Each of the following responses was given once: 1 year, 3 years, 4 years, 5 years, 10 years, no answer.

**Four of these respondents said their journals stored electronic versions.

Consent forms were available (downloadable from their websites or distributed by editors) from 15 journals, eight of which accepted only their own forms, while six also accepted other forms if they contained all necessary items. Of the journals that did not provide their own consent forms, five confirmed the contents of submitted consent forms (Table 4).

**Table 4:** Editors’ reports on their journals’ other practices regarding consent forms

**Questions**	**Number of responses**	**Percentage**
Are consent forms available from the journal?		
Yes (form can be downloaded from the website)	14	60.9
Yes (form must be requested from an editor)	1	4.3
No	8	34.8
Does the journal accept other consent forms?		
*This question targeted those who responded “yes” to the first question (n = 15).*		
Yes, if they were developed by researchers or research institutions	1	6.7
Yes, if they were developed by researchers or research institutions and include all necessary items*	6	40.0
No	8	53.3
Does the journal confirm the contents of submitted consent forms?		
*This question targeted those who responded “no” to the first question (n = 8).*		
Yes	5	62.5
No	3	37.5

*Note:* Survey responses represented 23 journals.

*Respondents identified the following items as necessary (multiple answers possible): consent for publication (n = 4); inclusion of identifying information (n = 3); acceptance of consent forms written in other languages (n = 2); objective of the study (n = 2); protection of subject’s identity (n = 1); subject’s opportunity to review the manuscript (n = 1), other (n = 1).

**Figure 2:**
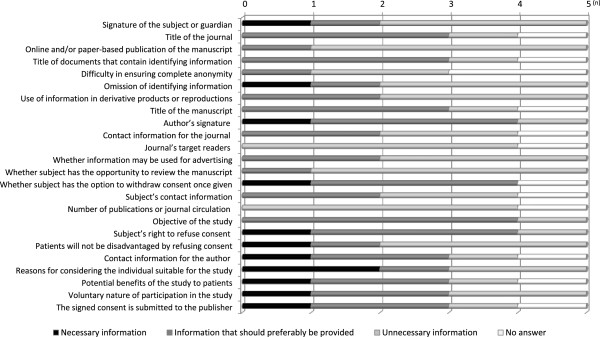
**Journal editors’ views on items that should be included on consent forms.** These questions targeted survey respondents who indicated that their journals did not provide consent forms and that they checked the contents of submitted consent forms (n = 5).

To examine whether the specific items included in journals’ consent forms are necessary for editors whose journals did not provide consent form, we asked questions regarding the need for each item to five editors. Of these, three regarded the following consent-form items as “necessary information” or “information that should preferably be provided”: title of the journal, title of documents that contain identifying information, title of the manuscript, signature of the author, withdrawal of consent, purpose of the study, subject’s right to refuse consent, contact information for the author, reasons for considering the individual suitable for the study, potential benefits of the study to patients, voluntary nature of participation in the study, and the information that signed consent forms would be sent to the publisher. The following items were regarded as unnecessary by three or more journals: signature of subject or guardian, online or paper-based publication of the manuscript, omission of identifying information, use of information in derivative products or reproductions, target readers of the journal, use of information for advertising purposes, whether subjects were allowed to review the manuscript, number of publications, circulation of the journal, and assurance that patients would not be disadvantaged by refusing consent for publication of their information (Figure 2).

In addition, some comments were made on the management of consent forms. Considering problems possibly occurring several years after publication (for example, subjects who were children during the study may object, after they grow up, to the publication of their identifying information), one editor pointed out the necessity of a permanent record of consent, while another considered obtaining consent particularly challenging in the field of psychiatry. It was also pointed out that consent for use of data for research and educational purposes is insufficient, and it is particularly important to obtain consent for publication of a case description or clinical photograph.

## Discussion

This study examined current practices in case reports and other studies publishing identifying information, with the hope of identifying issues around obtaining consent.

Obtaining consent or consent forms was considered necessary by approximately 40% of journals under review, and the submission of consent forms was required by 30% of the journals. Approximately 10% provided their own consent forms. While there may be differences between specialties and publishing groups in each field, these rates are higher than those reported in a previous study [21], suggesting an increasing interest in case reports and studies publishing identifying information.

The frequency of statements regarding obtaining consent varied depending on the specialist field. Statements were found most frequently in pediatrics journals (58.0%), and least frequently in genetics and heredity journals (21.1%). Similarly, statements regarding substitute consent were found most frequently in pediatrics journals (29.0%), and least frequently in genetics and heredity journals (10.9%). Specific mentions of pedigrees in instructions for authors were found least frequently in genetics and heredity journals (2.0%). With genetics being a field in which psychological and financial (insurance and employment) risks have been highlighted in discussions of the need to consider the influence on subjects and their families of publication [8], special attention is required when obtaining consent for the publication of pedigrees. Similarly, in the field of pediatrics, in which it has been considered more appropriate, but is actually difficult to obtain consent when subjects grow up sufficiently to make their own judgments [10], instructions for considering the influences of publication on a child’s future were observed in only one journal. To address this problem, some journal editors responding to the questionnaire pointed out the need to store consent forms for a longer period of time. There were also statements regarding mental impairment in some journals; in a previous study [10], substitute consent was not regarded as appropriate for subjects with impaired judgment. Despite the need for sensitivity in this area, few of the journals covered by this study clearly presented their policies.

In the consent forms provided by journals, a large number of statements were found regarding subjects’ signatures, journal names, non-paper-based publication, and how it would not be possible to completely anonymize subject data. These were regarded as necessary contents. The ICMJE [6] recommends that authors disclose to their subjects whether the manuscript includes identifiable personal data available online, and 26 out of 27 journals’ consent forms adhered to this requirement. As described in the Declaration of Helsinki [22], which requires that potential subjects must be informed of any potential risks involved in taking part in a study, more than 80% of consent forms described the risk of the possible identification of the subject. However, statements about the study objective, subjects’ right to refuse consent, and provisions for withdrawal of consent were found infrequently, although these points were considered to be important by editors responding to the questionnaire. Thus, there is a clear inconsistency between the contents of existing consent forms and opinions expressed by survey respondents about what those forms should contain. The study objective, subjects’ unconditional right to refuse consent, and withdrawal of consent at any time without reprisal, were considered as necessary contents for informed consent in the Declaration of Helsinki [22]. Similarly, in studies that publish identifiable personal information, explanation of the study’s scientific or medical significance may determine whether a subject consents to their information being published. Giving assurance of subjects’ right to refuse consent may also give rise to the need for the subject to be able to give consent discretionally. As some journals provided information about withdrawal of consent, providing this information on withdrawal of consent may affect a subject’s decision to give consent, and the need to add this to the forms is suggested.

Problems with consent forms were also pointed out in some previous studies. To avoid the necessity of repeatedly obtaining consent when manuscripts are rejected by journals accepting only their original consent form, the development of common forms has been proposed [11,12,19]. However, a large number of editors responding to the questionnaire said that their journals required the submission of consent forms with manuscripts, and editors from eight of the journals providing a consent form stated that they did not accept other forms. Only a few of the journals reviewed for this study provided multilingual consent forms. Difficulty in using such forms by non-native English speakers was pointed out in a previous study [23]. It is suggested therefore that several issues remain unresolved.

Although the ICMJE [6] recommends that journals should establish their own policies with local legal guidance, they also recommend that consent forms should be archived with the journal, the authors, or both. The criticism that subjects’ identifiable personal data are transferred to the journal by the submission of signed consent forms has been raised [13]. On this issue, the ICMJE suggests that, to protect patient confidentiality, journals may decide that the author archives the consent form and provides the journal with a written statement that they have received and archived written patient consent. In our study, approximately 30% of journals required submission of a signed consent form, and 56.5% of journals stored submitted consent forms indefinitely. In their report on patient confidentiality and publishing, Bal et al [13] state that consent forms should be explicit that identifiable personal information will be transferred to a journal for publication. One journal required statements about the submission of signed consent forms to the publisher as part of the consent form. However, a statement about submitting signed consent forms to the publisher was not found in half of journals’ consent forms reviewed in our study, despite including statements about submission of consent forms in their instructions for authors. We suggest that it is possible that subjects may not notice or be made sufficiently aware that their information will be transferred to a journal for publication.

The questionnaire results indicated that the most common reason for requiring such a submission was to confirm that authors had obtained consent. To confirm the contents of the consent form was listed as the reason in fewer than half the journals reviewed in our study. In current practice, archiving signed consent forms is recommended by the ICMJE [6], and it is expected that the journal producing its own consent forms and requiring submission of signed consent forms is a way to ensure research ethics are complied with. In practice however, the issue of consent is between the author and the subject, and whether the publisher should take responsibility for subject consent, beyond supporting author’s ethical practice, requires further discussion. Although it may be viewed that author’s declaration to journals that they have obtained consent is sufficient [13], it is necessary to discuss the roles of editors and researchers in guaranteeing ethics.

Regarding points to consider when obtaining consent, statements allowing subjects to review manuscripts before publication or regarding non-paper-based publications were occasionally noted. Statements advising consideration of the effects on subjects of publishing identifying information were rarely observed. Examples of the difficulty of maintaining anonymity appeared in some consent forms; however, it is questionable whether such examples alone are enough to lead subjects to consider the effects on themselves. Case reports and studies publishing identifying information mainly differ from major clinical and epidemiological studies in the possibility of individual identification and consequent effects on subjects and their families. Studies have pointed out the necessity of explaining benefits and risks in detail when obtaining consent [5] and the presence of uncontrollable risks after consenting (inappropriate use of information and media follow-up) [20]. As such effects depend on subjects’ backgrounds, it may be necessary for authors or researchers to implement different consent procedures appropriate to each case.

Difficulty has been reported in obtaining consent from individuals with impaired judgment, such as children and patients with mental disorders, and in cases of medical malpractice, unavailability of subjects, their deaths, and possible interference with the physician-patient relationship [10,14]. In line with this, obtaining consent has not been regarded as absolutely necessary on some occasions [15,16], while other researchers have insisted on the necessity of considering it even when it is difficult [5] and of giving importance to the decision-making process [17]. It has also been pointed out that relatives’ consent should be obtained after a subject’s death [18]. On analysis of instructions for authors, statements that identifying information should be deleted when consent is not available were found. In fact, obtaining consent is needed because complete anonymity is impossible. Therefore it is necessary to include only scientifically necessary information and maintain anonymity of identifiable information [24], and furthermore, to determine whether to obtain consent or to tolerate publishing without consent despite potential identifiability. Although these points interact with each other, further discussion may be necessary to focus separately on issues such as the content of consent and points to consider when obtaining it, and the appropriateness of obtaining or not obtaining consent.

This study had several limitations. The review of instructions for authors studied only those texts and, to a limited degree, linked documents, and therefore may not have reflected journals’ policies as described elsewhere, although it is likely to have discovered the most important items. In addition, the questionnaire survey of journal editors was not anonymous, to allow comparison of editors’ responses with their journal’s instructions for authors, observation of any differences between fields. However, between-field comparisons could not be accomplished because the response rate was low. Furthermore, it may be difficult to generalize its results, considering the possible influences of the questionnaire’s design on editors’ responses and response rate. The limited number of journals under study also did not necessarily represent general tendencies among academic journals. However, these surveys focused on key fields in which consent-related problems have been pointed out, while taking diversity into consideration.

## Conclusion

Consent was regarded as necessary by approximately 40% of the journals under study, and the submission of consent forms was required by 30%. Approximately 10% of journals provided their own consent forms. However, differences were observed according to the field. Specific items regarding publishing identifiable personal information were not always described. Although the difficulty in achieving complete anonymity was described as a risk in the journals’ consent forms studied, we believe it is necessary to also include items such as the study objective, subjects’ right to refuse consent, and withdrawal of consent on the forms. The main reason stated for journals asking for the submission of a signed consent form was to confirm that consent had been obtained, but further discussion is needed on whether the responsibility to confirm this lies with the publisher. In this study, the investigation of current practice among publishers was performed. However, further study is needed into researchers who obtain the consent in practice, and subjects who give their informed consent. In case reports and other studies publishing identifying information, it is expected that appropriate consent procedures will need to be examined in more detail in the future.

## Competing interests

The authors declare that they have no competing interests.

## Authors’ contributions

AY conceived the study, participated in its design and coordination, and helped to draft the manuscript. SK conceived the study, participated in its design and coordination, and helped to draft the manuscript. YD and HM performed the content analysis. All authors read and approved the final manuscript.

## Pre-publication history

The pre-publication history for this paper can be accessed here:

http://www.biomedcentral.com/1472-6939/14/47/prepub

## Supplementary Material

Additional file 1 Questionnaire for journal editors. Click here for file
