# Supplementary material for: Obtaining subjects’ consent to publish identifying personal information: current practices and identifying potential issues
Source: BMC Med Ethics. 2013 Nov 25;14:47. doi: 10.1186/1472-6939-14-47 (PMC4222768; doi:10.1186/1472-6939-14-47)
Supplement: Additional file 1 — Questionnaire for journal editors. [file 1472-6939-14-47-S1.pdf]

Additional file 1

Questionnaire for journal editors

1. What is the title of the journal? ( )

2. When is an author supposed to submit a written consent form?

☐ At the time of submission of a manuscript

☐ At the time of acceptance of a manuscript

☐ Between the acceptance of a manuscript and its publication

☐ Other timing ( )

3. Until when do you store a written consent form?

☐ We discard it as soon as we confirm that the author has acquired it.

☐ We store it until a decision is made on the acceptance or rejection of a manuscript, and then discard it.

☐ We store it until the case report is published.

☐ We store it for a certain period after publication, and then discard it.

How long do you store a written consent form? ( )

☐ Others ( )

4. Why do you require author to submit a written consent form? (Multiple answers allowed)

☐ To confirm the acquisition of written consent

☐ To confirm the items in the written consent form

☐ Other purposes ( )

5. Is your original consent form available?

☐ Yes (The form can be downloaded from the website.)

☐ Yes (An author is required to ask the editor for the form.)

☐ No

6. (Question5: Yes) Do you accept consent forms written in formats other than the specified one?

☐ No, we do not accept consent forms written in other formats.

☐ Yes, we accept consent forms originally created by researchers or research organizations.

☐ Yes, we accept consent forms originally written by researchers or research organizations, provided that they include necessary items.

Please specify the necessary items. ( )

☐ Others ( )

7. (Question5: No) Do you examine the items provided in the consent form?

☐ Yes

☐ No

8. (Question7: Yes) Should the consent form provide the following information? Please check one of the three checkboxes for each item

	Required information	Information that should preferably be provided	Unnecessary information
1. Title of the journal	☐	☐	☐
2. Title of the manuscript	☐	☐	☐
3. Signatures of patients (or their guardians)	☐	☐	☐
4. Addresses, telephones, and e-mail addresses of patients	☐	☐	☐
5. Purpose of the study	☐	☐	☐
6. Title of documents that are identifiable or used	☐	☐	☐
7. Reasons for considering the individual suitable for the study	☐	☐	☐
8. The mention of the potential benefits of the study to patients	☐	☐	☐
9. Omission of identifiable information	☐	☐	☐
10. An explanation that it is difficult for patients to remain completely anonymous	☐	☐	☐
11. Online and/or printed publication of the manuscript	☐	☐	☐
12. The number of publication / the circulation of journal	☐	☐	☐
13. Target readers of the journal	☐	☐	☐
14. An explanation of possible reproduction,	☐	☐	☐

reprint and use of material or information in the own journal, other licensed publication and derivative products			
15. An explanation that patients are allowed to view or check the manuscript	☐	☐	☐
16. The material or information may be used or not be used for advertising purpose	☐	☐	☐
17. Withdrawal of consent	☐	☐	☐
18. Voluntary participation of study	☐	☐	☐
19. Patients' right to refuse consent	☐	☐	☐
20. An explanation that patients will not be disadvantaged in any manner by refusing consent	☐	☐	☐
21. An explanation that the written consent is submitted to the publisher	☐	☐	☐
22. Signature of the author	☐	☐	☐
23. Contact information for the author	☐	☐	☐
24. Contact information for the journal	☐	☐	☐

Specify other items that you think should be included in a consent form.

( )

9. Please write your opinions, if any, about the submission of a written consent and handling of a submitted consent form.

( )
